# Tumor Microenvironment Analysis Identified Subtypes Associated With the Prognosis and the Tumor Response to Immunotherapy in Bladder Cancer

**DOI:** 10.3389/fgene.2021.551605

**Published:** 2021-03-01

**Authors:** Hongxian Zhang, Jiwen Song, Junqiang Dong, Zhuo Liu, Lixuan Lin, Bing Wang, Qiang Ma, Lulin Ma

**Affiliations:** ^1^Department of Urology, Peking University Third Hospital, Beijing, China; ^2^Department of Urology, Shanxi Provincial Cancer Hospital, Taiyuan, China; ^3^Department of Urology, Heze Municipal Hospital, Heze, China; ^4^Department of Biotechnology, College of Life Sciences, Sichuan University, Chengdu, China; ^5^Department of Oncology, Wuhan No.1 Hospital, Wuhan, China; ^6^Department of Oncology, People’s Hospital of Xintai City, Xintai, China

**Keywords:** tumor microenvironment, immune cells, bladder cancer, differentially expressed genes, somatic mutation, copy number variation, immune checkpoint inhibitors, overall survival

## Abstract

**Background**: The efficiency of immune checkpoint inhibitors (ICIs) in bladder cancer (BLCA) treatment has been widely validated; however, the tumor response to ICIs was generally low. It is critical and urgent to find biomarkers that can predict tumor response to ICIs. The tumor microenvironment (TME), which may play important roles to either dampen or enhance immune responses, has been widely concerned.

**Methods**: The cancer genome atlas BLCA (TCGA-BLCA) cohort (*n* = 400) was used in this study. Based on the proportions of 22 types of immune cells calculated by CIBERSORT, TME was classified by K-means Clustering and differentially expressed genes (DEGs) were determined. Based on DEGs, patients were classified into three groups, and cluster signature genes were identified after reducing redundant genes. Then TMEscore was calculated based on cluster signature genes, and the samples were classified to two subtypes. We performed somatic mutation and copy number variation analysis to identify the genetic characteristics of the two subtypes. Correlation analysis was performed to explore the correlation between TMEscore and the tumor response to ICIs as well as the prognosis of BLCA.

**Results**: According to the proportions of immune cells, two TME clusters were determined, and 1,144 DEGs and 138 cluster signature genes were identified. Based on cluster signature genes, samples were classified into TMEscore-high (*n* = 199) and TMEscore-low (*n* = 201) subtypes. Survival analysis showed patients with TMEscore-high phenotype had better prognosis. Among the 45 differentially expressed micro-RNAs (miRNAs) and 1,033 differentially expressed messenger RNAs (mRNAs) between the two subtypes, 16 miRNAs and 287 mRNAs had statistically significant impact on the prognosis of BLCA. Furthermore, there were 94 genes with significant differences between the two subtypes, and they were enriched in RTK-RAS, NOTCH, WNT, Hippo, and PI3K pathways. The Tumor Immune Dysfunction and Exclusion (TIDE) score of TMEscore-high BLCA was statistically lower than that of TMEscore-low BLCA. Receiver operating characteristic (ROC) curve analysis showed that the area under the curve (AUC) of TMEscore and tumor mutation burden (TMB) is 0.6918 and 0.5374, respectively.

**Conclusion**: We developed a method to classify BLCA patients to two TME subtypes, TMEscore-high and TMEscore-low, and we found TMEscore-high subtype of BLCA had a good prognosis and a good response to ICIs.

## Introduction

Bladder cancer (BLCA) is the tenth most common form of cancer worldwide, with an estimated 549,000 new cases and 200,000 deaths according to global cancer statistics in 2018 (Bray et al., 2018). BLCAs are biologically heterogeneous, and have different clinical outcomes and therapeutic responses (Knowles and Hurst, 2015). Molecular stratification of BLCAs may stratify patients for prognosis or response to treatment. Several molecular classifications of BLCAs have been reported, which have improved the clinical management of BLCA (Choi et al., 2014; Damrauer et al., 2014; Rebouissou et al., 2014; Lerner and Robertson, 2016; Hurst et al., 2017; Robertson et al., 2017; Marzouka et al., 2018; Mo et al., 2018; Kamoun et al., 2020).

At present, radical resection remains the mainstay treatment for localized BLCA, followed by intravesical chemotherapy or immunotherapy. However, the 5-year recurrence rate for non-muscle-invasive BLCA ranged from 50 to 70%, and that for muscle-invasive BLCA was around 50% (Cambier et al., 2016; Kamat et al., 2016; Babjuk et al., 2017). After trimodality therapy, the 5-year survival rate for muscle-invasive BLCA was 65–70% (Kamat et al., 2016; Mitin et al., 2016; Zhan et al., 2018). Immune checkpoint inhibitors (ICIs), owing to their marvelous and durable anti-tumor activity, have changed the treatment scenario of metastatic cancer. The efficiency of ICIs in BLCA treatment has been widely validated (Balar, 2017; Brower, 2017; Feld et al., 2019), however, the tumor response to ICIs was generally low (Zou et al., 2016; Sonpavde et al., 2018). Therefore, it is critical and urgent to find biomarkers that can predict tumor response to ICIs (Sonpavde et al., 2018). Programmed cell death protein ligand 1 (PD-L1) is a commonly used biomarker to predict the tumor response to ICIs treatment (Liu et al., 2018). However, the specificity of PD-L1 expression level in predicting ICI efficiency has been challenged (Munari et al., 2018). Another significant issue related to PD-L1 that remains to be addressed is the definition of a proper cutoff value (Zou et al., 2016). Tumor mutation burden (TMB) is an emerging biomarker to evaluate the efficacy of ICIs since it is correlated to neoantigens (Das, 2018; Hellmann et al., 2019). Similar to PD-L1, the breakpoint between TMB-high and TMB-low remains to be defined (Samstein et al., 2019). Microsatellite instability (MSI) is another biomarker of the efficiency of ICIs (Overman et al., 2018), but it is only applicable to a few types of tumors, such as colorectal cancer (Overman et al., 2017). Thus, a single biomarker may not be sufficient to predict the efficacy of immunotherapy.

The tumor microenvironment (TME) is the battlefield where tumor cells confront host immune system directly. Several studies have explored the relationship between TME subtypes and tumor response to ICIs, and the role of tumor-infiltrating lymphocytes (TILs) and cytokines in immunotherapy has been demonstrated in a variety of tumors (Zeng et al., 2019). Nevertheless, the effect of TME on the tumor response to immunotherapy in BLCA is still under-investigated. Recently, Pfannstiel et al. (2019) published a comprehensive report on the role of stromal TILs in the prognosis of 542 cases with muscle-invasive BLCA, but immunocytes other than TILs were not included, which are also important for the formation of TME.

The purpose of this study was to identify the TME subtypes of BLCA with different biological behaviors. We also investigated the correlation between the TME subtypes and tumor response to immunotherapy in BLCA as well as the clinical outcome of BLCA.

## Materials and Methods

### Study Cohort

**Figure 1:**
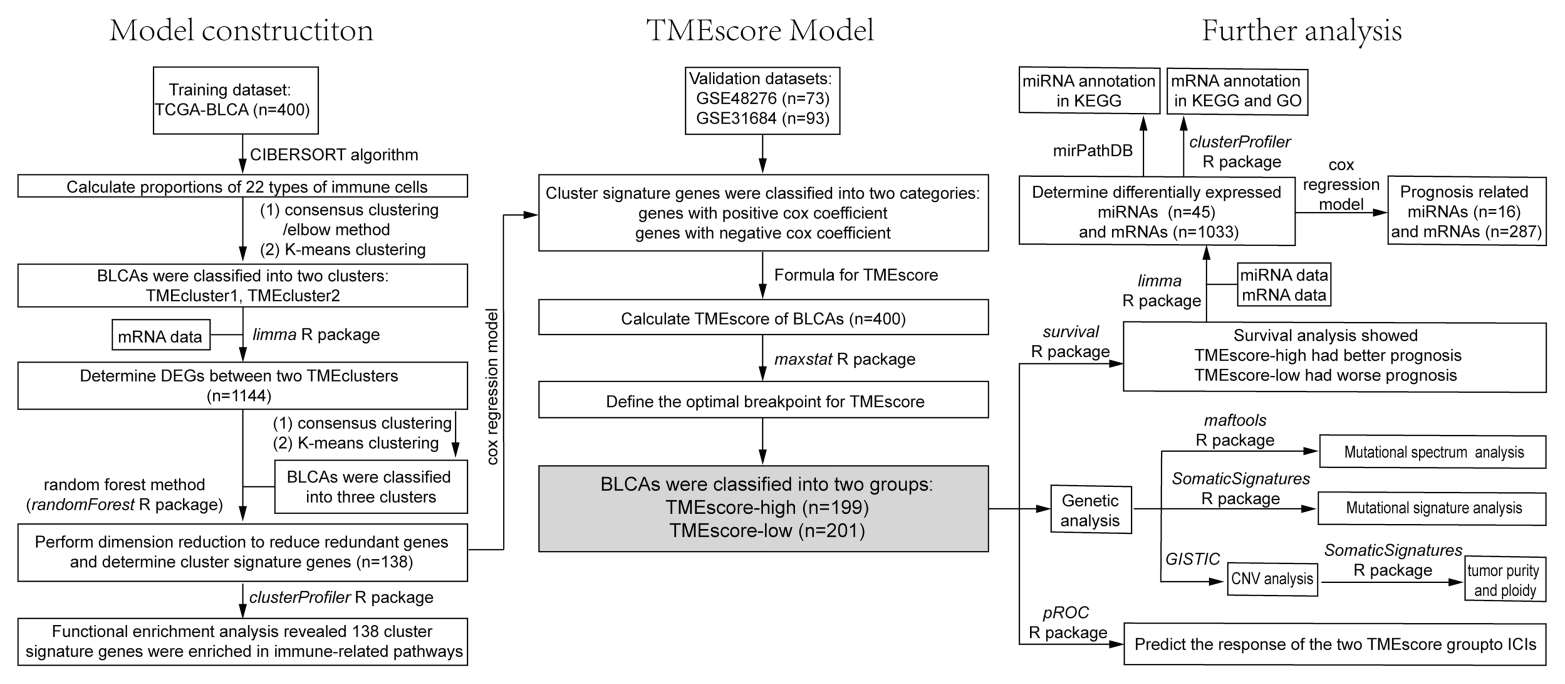
The flowchart of the model construction process and further analysis, including survival analysis, correlation analysis, and genetic analysis.

The Cancer Genome Atlas bladder cancer (BLCA) cohort (TCGA-BLCA) was used in this study, including 430 samples with RNA-seq data, 411 samples with single nucleotide variant (SNV) data, 413 samples with copy number variant (CNV) data, and 429 samples with micro-RNA (miRNA) data. BLCA samples (*n* = 400) with both RNA-seq data and clinical information were used for further analysis, including patients with Stage I (*n* = 2), Stage II (*n* = 128), Stage III (*n* = 138), and Stage IV (*n* = 130). Data sets GSE48276 (*n* = 73) and GSE31684 (*n* = 93) downloaded from GEO were used as validation sets. The information of data sets was summarized in Supplementary Table 1. The flowchart of this study was shown in Figure 1.

### TME Analysis

Based on the RNA-seq data of 400 BLCA samples, the proportions of 22 types of immune cells were calculated by the CIBERSORT algorithm (Newman et al., 2015). Unsupervised hierarchical clustering of immune cells was performed to define cell clusters based on the proportions, and the correlation of each immune cell with other immune cells as well as the correlation with survival was analyzed. The TME cell network was plotted by Cytoscape (Shannon et al., 2003). According to the proportions of immune cells, BLCA samples were grouped using different methods (elbow method or consensus clustering) and the optimum K was determined. Then, TME patterns were identified by K-means clustering and patients were classified. Differentially expressed genes (DEGs) among these classes were determined using *limma* R package at thresholds of adjusted value *p* < 0.05 and |log2FC| > log2(1.5) (Ritchie et al., 2015). Subsequently, based on the DEGs, patients were clustered using the *ConsensusClusterPlus* R package, and the clusters were obtained by K-means clustering. Finally, the cluster signature genes were obtained after reducing redundant genes by random forest method using *randomForest* R package (Kursa and Rudnicki, 2010), and the enrichment analysis was performed on the cluster signature genes using the *clusterProfiler* R package.

Based on cox regression model, the cluster signature genes were classified into two categories according to cox coefficient (positive or negative; Sotiriou et al., 2006). TMEscore was calculated as follows:

$$TMEscore=\sum \log_{2}\left(X+1\right)-\sum \log_{2}\left(Y+1\right)$$

*X* represents the expression value of cluster signature genes with a positive cox coefficient, and *Y* represents the expression value of cluster signature genes with a negative cox coefficient.

The *maxstat* R package was used to define the optimal breakpoint for TMEscore, thus samples can be classified to TMEscore-high and TMEscore-low subtypes.

### Survival Analysis and Correlation Analysis

Survival R package was used for survival analysis to analyze the correlation between TMEscore subtypes and clinical outcomes. Survival curves were plotted using *survimer* R package. The differentially expressed miRNAs and messenger RNAs (mRNAs) between TMEscore-high and TMEscore-low subtypes were determined using *limma* R package, and the enrichment analysis of miRNAs and mRNAs was performed using miRPathDB and *clusterProfiler* R package, respectively. Based on cox regression model, prognosis related miRNAs and mRNAs were identified, and the survival analysis of these miRNAs and mRNAs was performed.

The correlation between TMEscore and tumor response to ICIs in BLCA was explored. Tumor Immune Dysfunction and Exclusion (TIDE) scoring system was used to evaluate tumor response to ICIs, in which the higher the TIDE score, the worse the tumor response to ICIs and the worse the prognosis (Jiang et al., 2018). Kaplan-Meier method was used to analyze overall survival (OS) stratified by TME score. Statistical significance was defined as two-tailed values *p* < 0.05.

### Somatic Mutation and Copy Number Variation Analysis

We performed somatic mutation analysis based on 400 BLCA samples. Mutational spectrum and mutational signature were depicted *via maftools* and *SomaticSignatures* R packages, respectively. Significant chromosomal regions harboring CNVs were identified by GISTIC. Based on the results of CNVs, tumor purity and ploidy were estimated by *ABSOLUTE* R package. Furthermore, a landscape of molecular and clinical characteristics for two TME subtypes in BLCA was depicted.

## Results

### TME Subtypes are Associated With the Prognosis of BLCA

**Figure 2:**
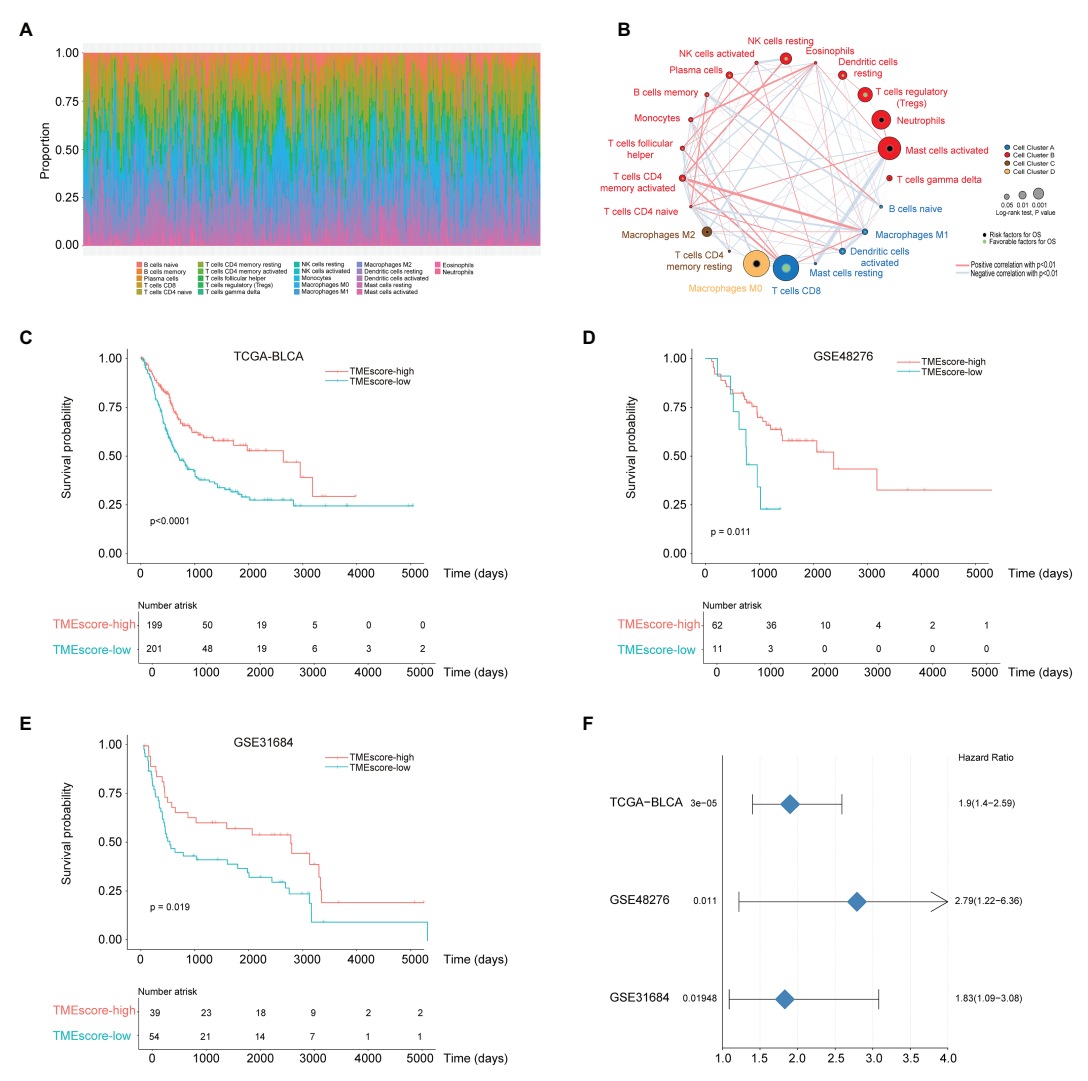
The tumor microenvironment (TME) subtypes are associated with the prognosis of bladder cancer (BLCA). **(A)** The proportions of 22 types of immune cells. **(B)** The cellular interactions of 22 types of immune cells and their correlations with overall survival (OS). Cell cluster A, blue; Cell cluster B, red; Cell cluster C, brown; and Cell cluster D, orange. The size of the circle indicates the degree of correlation with the OS. Risk factors for OS are indicated in black, and favorable factors for OS are indicated in green. The thickness of the line indicates the degree of cellular interactions; the red lines indicate positive correlations, and the blue lines indicate positive correlations. **(C–E)** Kaplan-Meier curves for OS of the cancer genome atlas BLCA (TCGA-BLCA), GSE48276 and GSE31684 cohort, respectively. **(F)** The forest plot for survival analysis of different data sets.

The proportions of 22 types of immune cells were presented in Figure 2A. Based on the proportions, immune cells were classified into four clusters using unsupervised hierarchical clustering (Supplementary Figure 1A). Immune cells in each cluster have similar functions. Immune cells in Cluster A recognize antigens and act as messengers between the innate and the adaptive immune systems, including activated dendritic cells, CD4 naïve T cells, resting mast cells, naïve B cells, CD8 T cells, and macrophage M1. Most immune cells in Cluster B have the function of attacking and killing exogenous antigen, such as activated NK cells, T cells regulatory, T cells follicular helper, plasma cells, neutrophils, and activated mast cells. Cluster C includes macrophage M2, which decrease inflammation and encourage tissue repair, and CD4 memory resting T cells. Non-activated macrophage M0 forms Cluster D alone. Pearson’s correlation coefficients were calculated to investigate the correlation between different immune cells, and the correlation between immune cells and survival was analyzed using survival R package. Cellular interactions of immune cells and their correlations with OS were visualized using Cytoscape (Figure 2B). The TME cell network revealed that four types of immune cells, macrophages (M0), CD8^+^ T cells, mast cells (activated), and neutrophils, had significant effects on the prognosis of BLCA (*p* < 0.05). Among them, CD8^+^ T cells, mast cells (activated) and neutrophils were positively correlated with OS, and macrophages (M0) were negatively correlated with OS. Based on the proportions of immune cells, elbow method and consensus clustering were applied to identify the optimal K value to classify TME patterns, and as a result two clusters were determined (*K* = 2; Supplementary Figures 1B–K). BLCA samples were then classified into two clusters using K-means clustering (Supplementary Table 2). The proportions of 22 types of immune cells varied between the two clusters (TMEcluster1 and TMEcluster2; Supplementary Figure 1L), and the OS was significantly different (*p* = 0.05) between them (Supplementary Figure 1M).

To identify the key factors associated with different clusters, we used the *limma* R package to find out DEGs between TMEcluster1 and TMEcluster2. As a result, there were 1,144 DEGs contributed to the TME classification. Based on these 1,144 DEGs, consensus clustering was used to determine the optimal K value (*K* = 3), and K-means clustering was conducted to classify patients into three classes (Supplementary Figure 2). After reducing redundant genes by random forest method, 138 cluster signature genes were obtained. Functional enrichment analysis (GO annotation) by *clusterProfiler* R package revealed that cluster signature genes were enriched in immune-related pathways. Based on cox regression model, the cox coefficient for each cluster signature gene was obtained and used to calculate TMEscore. As a result, the samples were classified into TMEscore-high (*n* = 199) and TMEscore-low (*n* = 201) subtypes (*x* = 0.035).

Survival analysis stratified by TMEscore showed that patients with TMEscore-high phenotype had better prognosis than those with TMEscore-low phenotype (Figure 2C; *p* < 0.0001). The model of TMEscore was tested using validation datasets GSE48276 and GSE31684. The results suggested that the model was credible and TMEscore-high was associated with good prognosis of BLCA (Figures 2D–F). DEG analysis obtained 45 differentially expressed miRNAs (Supplementary Figures 3A,B) and 1,033 differentially expressed mRNAs (Supplementary Figures 4A,B) between TMEscore-high and TMEscore-low subtypes, with the threshold adjusted value *p* < 0.01 for miRNA, and adjusted value *p* < 0.01 and |logFC| > 1 for mRNA, respectively. Differentially expressed miRNA annotation in KEGG using mirPathDB revealed several cancer related miRNAs, such as hsa-miR-200b-3p, hsa-miR-200a-3p, hsa-miR-200c-3p, and hsa-miR-429 (Supplementary Figure 3C). Annotation in GO and KEGG using *clusterProfiler* R package showed that differentially expressed mRNAs mainly enriched in the activation and regulation of immune response (Supplementary Figures 4C, 7) as well as remodeling of extracellular matrix (Supplementary Figure 4D).

**Figure 3:**
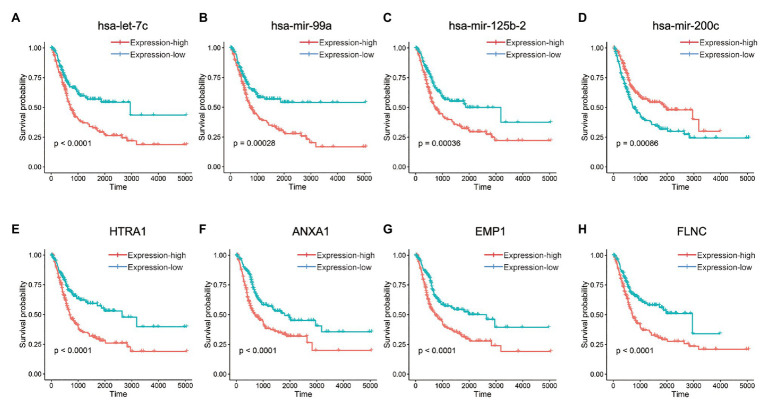
Kaplan-Meier curves for OS of differentially expressed micro-RNAs (miRNAs) and messenger RNAs (mRNAs) between TMEscore-high and TMEscore-low subtypes, which had statistically significant impact on the prognosis of BLCA. **(A–D)** The top four miRNAs. **(E–H)** The top four mRNAs.

Based on cox regression model, 16 differentially expressed miRNAs and 287 differentially expressed mRNAs were identified to have statistically significant impact on the prognosis of BLCA, among which the top four miRNAs were hsa-let-7c, hsa-mir-99a, hsa-mir-125b-2, and hsa-mir-200c (Figures 3A–D), and the top four genes were *HTRA1*, *ANXA1*, *EMP1*, and *FLNC* (Figures 3E–H). We performed unsupervised hierarchical clustering on 287 survival-related genes, and patients were classified into TMEscore-high and TMEscore-low groups (Supplementary Figure 5).

### Genetic Characteristics of TMEscore-High and TMEscore-Low Subtypes of BLCA

**Figure 4:**
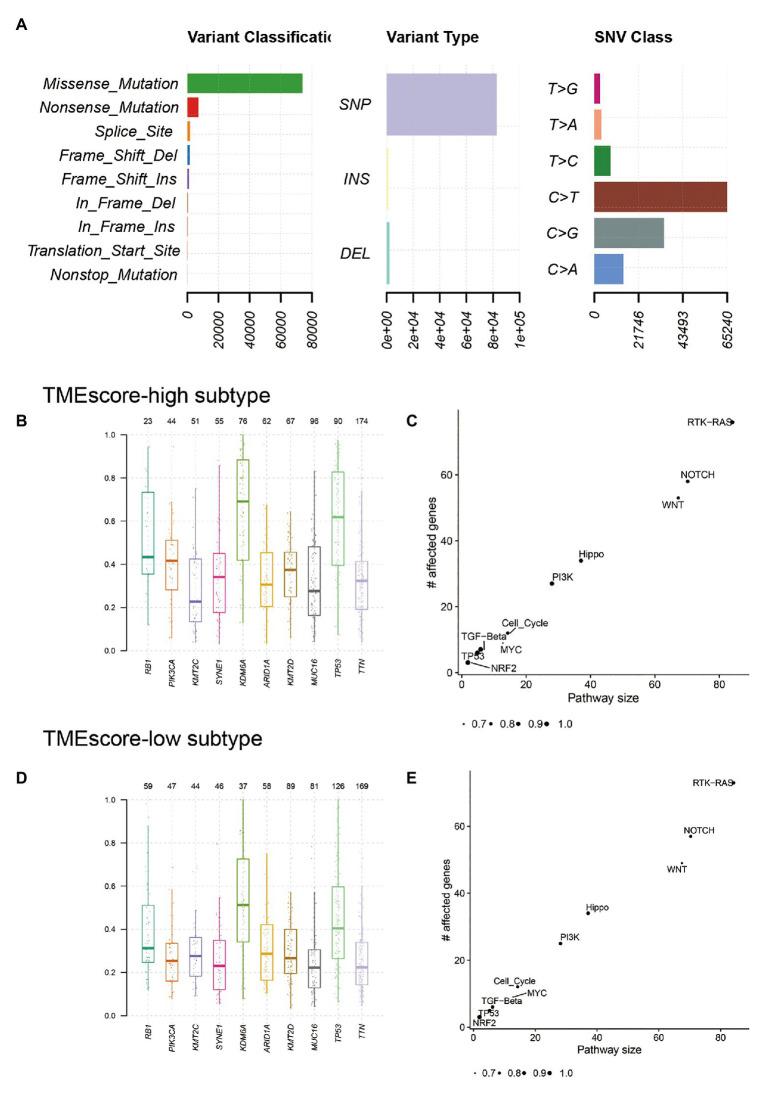
Genetic characteristics of TMEscore-high and TMEscore-low subtypes of BLCA. **(A)** The landscape of somatic mutations of BLCA. The variant allele fractions (VAFs) of top 10 frequently mutated genes in TMEscore-high subtype **(B)** and TMEscore-low subtype **(D)**. The enrichment analysis of differential genes in TMEscore-high subtype **(C)** and TMEscore-low subtype **(E)**.

The landscape of somatic mutations of BLCA was presented in Figure 4A. Missense mutation mainly caused by SNV was the major type of mutations, and C > T was the major type of base substitution. The most frequently mutated genes were *TTN* and *TP53* in TMEscore-high subtype and TMEscore-low subtype, respectively (Supplementary Figure 6). There were 94 genes with significant differences in mutation frequencies between TMEscore-high and TMEscore-low subtypes (*p* < 0.05), among which top 18 differential genes were *RB1*, *KDM6A*, *FGFR3*, *ELF3*, *TP53*, *KMT2A*, *NFE2L2*, *DZIP1*, *POLR2A*, *ALMS1*, *EP300*, *PIK3CA*, *SOX5*, *LRRC37B*, *PCDHB12*, *PPFIA2*, *ZNF462*, and *STAG2* (*p* < 0.01). Among the top 10 frequently mutated genes in BLCA, the variant allele fractions (VAFs) of *RB1*, *KDM6A*, *TP53*, *PIK3CA*, and *KMT2D* were significantly different between two subtypes (*p* < 0.05; Figures 4B,D). The KEGG pathway enrichment analysis of differential genes showed that RTK-RAS, NOTCH, WNT, Hippo, and PI3K signaling pathways were mainly altered in both TME subtypes (Figures 4C,E).

**Figure 5:**
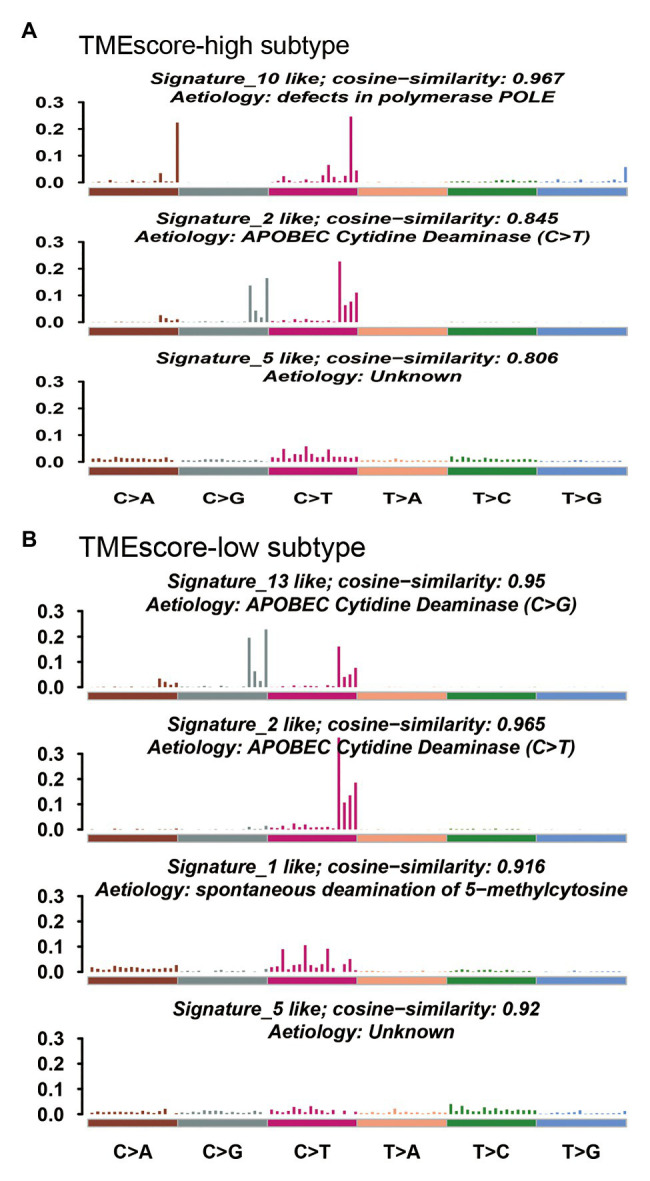
Mutational signatures associated with TMEscore-high subtype **(A)** and TMEscore-low subtype **(B)**.

Mutational signature analysis (Alexandrov et al., 2013) showed that TMEscore-high subtype was associated with Signature 2, Signature 5, and Signature 10, whereas TMEscore-low subtype was associated with Signature 1, Signature 2, Signature 5, and Signature 13 (Figure 5). Signature 1 is associated with spontaneous deamination of 5-methylcytosine; Signature 2 and Signature 13 are associated with APOBEC cytidine deaminase; and Signature 10 is associated with defects in polymerase POLE.

The CNVs analysis by GISTIC showed that amplifications of chromosomal arms 20q, 8q, and 20p, and deletions of chromosomal arms 9q, 9p, 8p, 11p, 5q, and 11q frequently occurred in TMEscore-high subtype (Supplementary Figure 7A); amplifications of chromosomal arms 20q, 8q, 20p, and 3q, and deletions of chromosomal arms 8p, 5q, 15q, and 17p frequently occurred in TMEscore-low subtype (Supplementary Figure 7B). The minimal common region (MCR) analysis showed that amplifications of 20p13, 17q12, and 5q11.2, and deletions of 10q21.1, 10q21.3, and 12p13.33 frequently occurred in TMEscore-high subtype (Supplementary Figure 7C); amplifications of 20p13, 5q11.2, and 4p16.1, and deletions of 10p11.22, 10q11.23, and 11p15.5 frequently occurred in TMEscore-low subtype (Supplementary Figure 7D). As shown, the two subtypes shared most amplification regions, but the deletion regions were different. Based on the results of CNV, the estimated tumor purity ranged from 0.33 to 1.00 and the estimated tumor ploidy ranged from 1.98 to 10. The tumor purity in TMEscore-high subtype was higher than that in TMEscore-low subtype, indicating lower tumor content in TMEscore-low subtype (Supplementary Figure 7E). However, the tumor ploidy was comparable between two subtypes, indicating that CNV might be a universal phenomenon in BLCA (Supplementary Figure 7F). There was significant difference in tumor purity between TMEscore-high and TMEscore-low subtypes (*p* = 0.027), but no significant difference in tumor ploidy.

### TMEscore-High subtype is Associated With Tumor Response to Immunotherapy in BLCA

**Figure 6:**
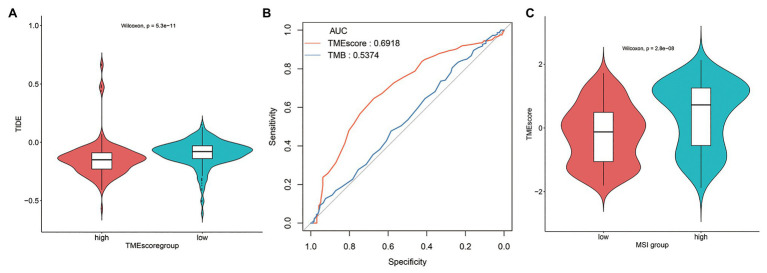
TMEscore-high subtype is associated with tumor response to immunotherapy in BLCA. **(A)** The tumor immune dysfunction and exclusion (TIDE) score of TMEscore-high and TMEscore-low subtypes. **(B)** Receiver operating characteristic (ROC) curves of TMEscore and tumor mutation burden (TMB). **(C)** The correlation analysis between TMEscore and microsatellite instability (MSI) status.

The TIDE score of TMEscore-high BLCA was statistically lower than that of TMEscore-low BLCA (Figure 6A), indicating that TMEscore-high was associated with better tumor response to immunotherapy in BLCA. Compared with TMB, an emerging biomarker for predicting the efficacy of ICIs, the predictive efficiency of TMEscore was significantly better than that of TMB based on receiver operating characteristic (ROC) curve analysis, in which the area under the curve (AUC) of TMEscore is 0.6918 and the AUC of TMB is 0.5374 (*p* < 0.0001; Figure 6B). Furthermore, the correlation analysis between TMEscore and MSI status was performed. As a result, the TMEscore of samples with MSI-H was significantly higher than samples with MSI-L/MSS (Figure 6C), inferring that both TMEscore-high and MSI-H (Hause et al., 2016) were associated with good response to ICIs.

The proportion of immune cells in TMEscore-high and TMEscore-low groups is shown in Supplementary Table 3. TMEscore-high group had significantly higher proportions of activated dendritic cells, monocytes, T cells follicular helper, and regulatory T cells, while TMEscore-low group had significantly higher proportions of macrophage M0/M1/M2 and CD4 memory resting T cells. Of note, BLCA patients in TMEscore-low group had a much higher proportion of macrophages than TMEscore-high group (34.81 vs. 19.18%). According to the recently published literature (Joseph and Enting, 2019), macrophages play roles in suppressing adaptive immunosurveillance and create a tumor favoring microenvironment in BLCA, suggesting that BLCA with high proportion of macrophages may have a poor response to immunotherapy, which is consistent with our findings. Moreover, the proportions of immune cells involved in adaptive immunity, such as activated dendritic cells, follicular helper T cells, and regulatory T cells, are higher in TMEscore-high group.

## Discussion

Transcriptome profiling has been a major tool for BLCA subtype discovery. Based on the large-scale transcriptomic data, Rebouissou et al. (2014) identified a subtype of BLCA presenting a basal-like phenotype, which was associated with shorter survival and presented an activation of the epidermal growth factor receptor (EGFR) pathway, implying basal-like BLCAs were sensitive to anti-EGFR therapy. Damrauer et al. (2014) used the gene expression data to classify high-grade BLCAs into two subtypes, termed “luminal” and “basal-like,” which have characteristics of different stages of urothelial differentiation and have clinically meaningful differences in outcome. Choi et al. (2014) performed whole genome mRNA expression profiling and identified three subtypes of BLCA, termed “basal,” “luminal,” and “p53-like,” which have different sensitivities to frontline chemotherapy. Using RNA-seq data, Robertson et al. (2017) identified five subtypes, termed “luminal-papillary,” “luminal-infiltrated,” “luminal,” “basal-squamous,” and “neuronal,” which may stratify response to different treatments. Marzouka et al. (2018) developed a mRNA classifier based on tumor cell phenotypes defined by extensive IHC analyses and identified five subtypes, termed “urothelial-like,” “genomically unstable,” “basal/SCC-like,” “mesenchymal-like,” and “small-cell/neuroendocrine-like.” Mo et al. (2018) developed a classifier from 18 genes differentially expressed in various layers of the bladder urothelium and identified two distinct subtypes in BLCA designated as “basal” and “differentiated.” On the basis of these six classifications, Kamoun et al. (2020) defined a unified consensus subtyping system consisting of six subtypes termed “luminal papillary,” “luminal nonspecified,” “luminal unstable,” “stroma-rich,” “basal/squamous,” and “neuroendocrine-like,” which differ regarding underlying oncogenic mechanisms, infiltration by immune and stromal cells, and histological and clinical characteristics, including outcomes.

In this study, we focused on stratification of BLCAs in response to immunotherapy. Although ICIs are effective for advanced urothelial cancer including BLCA (Niglio et al., 2019), the objective response rate of BLCA to ICIs ranged from 24.4 to 31%, thus robust biomarkers are needed to predict tumor response to immunotherapy (Massard et al., 2016; Sharma et al., 2016; Plimack et al., 2017; Petrylak et al., 2018). PD-L1 expression level is a widely used biomarker for immunotherapy, but its effectiveness has been questioned (Zou et al., 2016). TMB is considered a promising biomarker to predict the response to ICIs in many types of tumors (Chan et al., 2019); however, the breakpoint between TMB-high and TMB-low remains to be well defined (Samstein et al., 2019). Considering the amounts of TILs were correlated with therapeutic response of tumors to ICIs, Pfannstiel et al. (2019) used sTILs along with tumor subtypes to stratify BLCA, and identified three different inflammatory phenotypes and a unique tumor evasion phenotype, all affecting patient outcomes. However, other immunocytes were not included, which are also important for the formation of TME. In our study, we investigated TME subtypes and their correlations with the prognosis of BLCA and the tumor response to ICIs in BLCA. Immune cells participated in the local immune reactions within a tumor mass can be roughly divided into two camps, anti-tumor camp and pro-tumor camp (Riera-Domingo et al., 2020). One type of immune cell may play different roles in different types of tumors. In this study, we found that CD8^+^ T cells, mast cells (activated), and neutrophils were positively correlated with OS, and macrophages (M0) were negatively correlated with OS.

We performed unsupervised clustering method to classify BLCA samples based on the proportions of 22 types of immune cells. As is known, determining the optimal number of clusters remains an open question in partitioning clustering. Consensus clustering has been widespread used in genomic studies (Monti et al., 2003). Șenbabaoğlu et al. (2014) compared different methods to estimate K value, and the results suggested that proportion of ambiguous clustering (PAC) was more accurate than other methods. In this study, we used consensus clustering to find the optimal *K* value. We found that when *K* = 2, the consensus matrix was the crispest (Supplementary Figures 1B–G), CDF plot showed a flat middle segment (Supplementary Figure 1H), and the value of PAC was minimum (Supplementary Figure 1J). Then, we used elbow method to find the optimal *K* value. Although a clear inflection point was not found in the elbow plot, the sum of squared error (SSE) decreases the most when *K* = 2 (Supplementary Figure 1K). Therefore, BLCA samples were classified into two clusters, TMEcluster1 and TMEcluster2. Based on the DEGs between TMEcluster1 and TMEcluster2, we used this criterion to determine the optimal *K* value and classify BLCA samples. As shown in Supplementary Figure 2J, SSE decreased dramatically when *K* = 2 or *K* = 3. The results of consensus clustering showed that the consensus matrix was also crisp when *K* = 3 (Supplementary Figures 2A–F), and the value of PAC for *K* = 3 was as low as that for *K* = 2 (Supplementary Figure 2I). According to the principle of grouping as detailed as possible, we selected *K* = 3 as the optimal K value and BLCA samples were classified into three clusters.

Finally, two TME subtypes, TMEscore-high and TMEscore-low, were identified based on this BLCA cohort. DEGs between TMEscore-high and TMEscore-low subtypes mainly enriched in the activation and regulation of immune response as well as remodeling of extracellular matrix, both of which have been proved to be associated with the clinical outcomes of cancer patients (Oudin et al., 2016; Salmon et al., 2019). Among these DEGs, 16 miRNAs and 287 mRNAs had statistically significant impacts on the prognosis of BLCA, among which the top four miRNAs were hsa-let-7c, hsa-mir-99a, hsa-mir-125b-2, and hsa-mir-200c, and the top four genes were *HTRA1*, *ANXA1*, *EMP1*, and *FLNC*. These DEGs may be potential prognostic biomarkers and therapeutic targets for BLCA.

Based on TIDE score, a gene expression biomarker to predict the clinical response to ICIs (Jiang et al., 2018), we evaluated the tumor response to ICIs in TMEscore-high and TMEscore-low subtypes of BLCA. The result revealed that TMEscore-high was associated with better tumor response to immunotherapy in BLCA. Furthermore, we compared the predictive efficiency of TMEscore to predict the tumor response to ICIs with that of TMB by performing ROC curve analysis. As a result, TMEscore (AUC = 0.6918) had a better predictive efficiency than TMB (AUC = 0.5374). The relatively low AUC values of TMEscore or TMB may be due to the criticism of AUC. It has been pointed out by researchers that although AUC is a popular statistical approach with a long history, there has been considerable criticism of it, which indicates that AUC is a poor metric for evaluating markers for disease diagnosis, screening, or prognosis (Pepe and Janes, 2008). The clinical biomarkers usually have low AUC values ranging from 0.5 to 0.7 according to the literatures (Gail, 2008; Louie et al., 2015). Since the model of TIDE was developed and validated based on melanoma patients treated with first-line anti-PD1 or anti-CTLA4, TMEscore can be used as a biomarker to predict the tumor response to anti-PD1 or anti-CTLA4, but whether TMEscore can predict the tumor response to other ICIs remains uncertain.

There were both similarities and differences in genetic characteristics between TMEscore-high and TMEscore-low subtypes. Although there were 94 differential genes, the altered pathways of the two subtypes were mainly concentrated in RTK-RAS, NOTCH, WNT, Hippo, and PI3K signaling pathways. The *PIK3CA* mutation has been reported to be associated with improved recurrence-free survival and cancer-specific survival in patients with BLCA (Kim et al., 2015). In our study, we found a higher VAF of *PIK3CA* in TMEscore-high group (0.42) compared with TMEscore-low group (0.25; *p* < 0.01), indicating that *PIK3CA* may be a reliable prognostic biomarker for BLCA. The CNV analysis showed that the two subtypes shared most amplification regions, but the deletion regions were different. Mutational signature analysis showed that both subtypes were associated with APOBEC cytidine deaminase. However, Signature 10, which is associated with defects in polymerase POLE, was significantly dominant in TMEscore-high subtype of BLCA. POLE plays an important role in chromosomal DNA replication. It is responsible for the leading-strand synthesis as well as recognition and removal of mismatch nucleotides by its proofreading capacity through the POLE exonuclease domain, which is crucial for the maintenance of replication fidelity (Guerra et al., 2017). The dysfunction of polymerase POLE leads to a high mutation burden and high neoantigen burden; therefore, tumors harboring deficient POLE have more active local immune response and a higher TMEscore.

There are some limitations in this study. First, the TME clustering and scoring pipeline was not tested in other types of tumors, therefore, we do not know whether this method is BLCA-specific or can be applied in pan-cancer scenario. Second, we did not explore the relationship of TME subtypes with molecular subtypes of BLCA. Despite these shortcomings, this study provided a novel way to predict the prognosis of BLCA and tumor response to immunotherapy in BLCA.

## Conclusion

We developed a method to classify BLCA patients to two TME subtypes, TMEscore-high and TMEscore-low, and we found TMEscore-high subtype of BLCA had a good prognosis and a good response to ICIs. TMEscore may be used as a biomarker to predict the prognosis of BLCA and the tumor response to immunotherapy in BLCA.

## Data Availability Statement

The original contributions presented in the study are included in the article/Supplementary Material, further inquiries can be directed to the corresponding authors.

## Author Contributions

HZ was responsible for the data analysis. JS was a major contributor in writing the manuscript. JD contributed to the revision. ZL contributed to data analysis. LL downloaded the data sets from the database. BW arranged the figures. QM directed the manuscript writing. LM put forward the idea and directed the data analysis. All authors contributed to the article and approved the submitted version.

### Conflict of Interest

The authors declare that the research was conducted in the absence of any commercial or financial relationships that could be construed as a potential conflict of interest.
