# Supplementary material for: Tumor Microenvironment Analysis Identified Subtypes Associated With the Prognosis and the Tumor Response to Immunotherapy in Bladder Cancer
Source: Front Genet. 2021 Mar 1;12:551605. doi: 10.3389/fgene.2021.551605 (PMC7957069; doi:10.3389/fgene.2021.551605)
Supplement: Supplementary file 1 [file Data_Sheet_1.docx]

**Supplementary Table 1** The basic information of samples

| **Series accession numbers** | **Platform used** | **No. of input patients** | **AJCC_Stage** | **Survival overcome** |
| --- | --- | --- | --- | --- |
| TCGA-BLCA | Illumina RNAseq | 400 | I: 2, II: 128,  III: 138, IV: 130 | OS |
| GSE48276 | Illumina HumanHT-12 WG-DASL V4.0 R2 expression beadchip | 73 | NA | OS |
| GSE31684 | [HG-U133_Plus_2] Affymetrix Human Genome U133 Plus 2.0 Array | 93 | NA | OS |

**Supplementary Table 2** Results of K-means clustering to classify TME patterns.

| BLCA sample | TMEcluster |
| --- | --- |
| TCGA-FD-A3SS-01A | 1 |
| TCGA-E7-A97P-01A | 1 |
| TCGA-GC-A3WC-01A | 1 |
| TCGA-DK-A1A3-01A | 1 |
| TCGA-XF-AAMJ-01A | 2 |
| TCGA-ZF-A9RL-01A | 1 |
| TCGA-DK-A3IL-01A | 1 |
| TCGA-E7-A6MF-01A | 1 |
| TCGA-BT-A20R-01A | 2 |
| TCGA-H4-A2HQ-01A | 1 |
| TCGA-GV-A3QH-01A | 1 |
| TCGA-E7-A4XJ-01A | 1 |
| TCGA-XF-A8HE-01A | 1 |
| TCGA-FD-A5BT-01A | 1 |
| TCGA-G2-AA3C-01A | 1 |
| TCGA-BL-A3JM-01A | 2 |
| TCGA-XF-AAN3-01A | 1 |
| TCGA-FD-A3SR-01A | 1 |
| TCGA-4Z-AA89-01A | 1 |
| TCGA-G2-A2EO-01A | 1 |
| TCGA-KQ-A41R-01A | 1 |
| TCGA-BT-A20V-01A | 1 |
| TCGA-DK-A2HX-01A | 1 |
| TCGA-YF-AA3M-01A | 2 |
| TCGA-GV-A3QF-01A | 1 |
| TCGA-XF-A9T3-01A | 2 |
| TCGA-BT-A20T-01A | 1 |
| TCGA-XF-AAN0-01A | 1 |
| TCGA-E7-A7DV-01A | 1 |
| TCGA-GV-A40E-01A | 1 |
| TCGA-GC-A3OO-01A | 2 |
| TCGA-XF-A9ST-01A | 2 |
| TCGA-GV-A3JX-01A | 1 |
| TCGA-FJ-A3ZF-01A | 1 |
| TCGA-YC-A89H-01A | 2 |
| TCGA-CF-A9FM-01A | 1 |
| TCGA-DK-A1AD-01A | 1 |
| TCGA-G2-A2EK-01A | 1 |
| TCGA-E5-A2PC-01A | 1 |
| TCGA-C4-A0F1-01A | 2 |
| TCGA-FD-A43N-01A | 1 |
| TCGA-BL-A13J-01A | 2 |
| TCGA-DK-A1AE-01A | 1 |
| TCGA-FD-A3SO-01A | 2 |
| TCGA-DK-A3IK-01A | 2 |
| TCGA-BT-A20X-01A | 1 |
| TCGA-ZF-AA52-01A | 2 |
| TCGA-SY-A9G0-01A | 1 |
| TCGA-GV-A3JW-01A | 2 |
| TCGA-FD-A3N6-01A | 2 |
| TCGA-CU-A5W6-01A | 1 |
| TCGA-DK-A2I4-01A | 1 |
| TCGA-DK-AA6M-01A | 1 |
| TCGA-4Z-AA7Y-01A | 1 |
| TCGA-DK-A3WY-01A | 1 |
| TCGA-E7-A3X6-01A | 1 |
| TCGA-2F-A9KP-01A | 1 |
| TCGA-DK-A3IQ-01A | 1 |
| TCGA-CF-A5U8-01A | 1 |
| TCGA-CU-A3QU-01A | 2 |
| TCGA-FD-A6TI-01A | 1 |
| TCGA-GU-A42P-01A | 1 |
| TCGA-FD-A3B3-01A | 1 |
| TCGA-YF-AA3L-01A | 1 |
| TCGA-DK-AA76-01A | 2 |
| TCGA-ZF-A9R2-01A | 1 |
| TCGA-GU-AATQ-01A | 2 |
| TCGA-FD-A5C1-01A | 1 |
| TCGA-DK-A2I1-01A | 1 |
| TCGA-UY-A9PB-01A | 1 |
| TCGA-DK-A3X1-01A | 1 |
| TCGA-DK-A1AC-01A | 1 |
| TCGA-FD-A62N-01A | 1 |
| TCGA-DK-AA75-01A | 1 |
| TCGA-BL-A5ZZ-01A | 2 |
| TCGA-4Z-AA86-01A | 2 |
| TCGA-XF-AAN2-01A | 1 |
| TCGA-BT-A2LD-01A | 1 |
| TCGA-ZF-A9R1-01A | 1 |
| TCGA-ZF-A9R9-01A | 1 |
| TCGA-DK-A6AV-01A | 1 |
| TCGA-XF-AAMY-01A | 2 |
| TCGA-GU-A763-01A | 1 |
| TCGA-FD-A3NA-01A | 1 |
| TCGA-CF-A47T-01A | 1 |
| TCGA-GC-A3BM-01A | 1 |
| TCGA-GD-A3OP-01A | 1 |
| TCGA-SY-A9G5-01A | 1 |
| TCGA-KQ-A41S-01A | 1 |
| TCGA-4Z-AA7M-01A | 1 |
| TCGA-FD-A3SJ-01A | 1 |
| TCGA-S5-A6DX-01A | 1 |
| TCGA-UY-A8OD-01A | 2 |
| TCGA-DK-A3IV-01A | 1 |
| TCGA-CF-A5UA-01A | 2 |
| TCGA-GD-A2C5-01A | 1 |
| TCGA-ZF-AA56-01A | 1 |
| TCGA-XF-AAMT-01A | 2 |
| TCGA-FD-A6TD-01A | 1 |
| TCGA-FD-A62O-01A | 1 |
| TCGA-BT-A2LB-01A | 1 |
| TCGA-FD-A3N5-01A | 2 |
| TCGA-G2-A3VY-01A | 1 |
| TCGA-DK-A1AA-01A | 1 |
| TCGA-XF-A9SY-01A | 2 |
| TCGA-DK-AA6Q-01A | 1 |
| TCGA-G2-A2EL-01A | 2 |
| TCGA-E7-A6ME-01A | 1 |
| TCGA-HQ-A2OF-01A | 1 |
| TCGA-BT-A20N-01A | 1 |
| TCGA-FD-A3SP-01A | 2 |
| TCGA-BT-A20P-01A | 1 |
| TCGA-GV-A40G-01A | 1 |
| TCGA-CF-A47S-01A | 1 |
| TCGA-XF-A9T2-01A | 2 |
| TCGA-ZF-AA4X-01A | 1 |
| TCGA-XF-AAMQ-01A | 1 |
| TCGA-DK-AA6W-01A | 2 |
| TCGA-KQ-A41O-01A | 1 |
| TCGA-S5-AA26-01A | 1 |
| TCGA-DK-A1A5-01A | 2 |
| TCGA-XF-A9SP-01A | 1 |
| TCGA-FD-A3SL-01A | 2 |
| TCGA-FD-A6TB-01A | 1 |
| TCGA-FD-A6TA-01A | 1 |
| TCGA-XF-A8HB-01A | 1 |
| TCGA-UY-A8OB-01A | 1 |
| TCGA-GU-A767-01A | 1 |
| TCGA-FD-A43U-01A | 2 |
| TCGA-ZF-AA5H-01A | 2 |
| TCGA-5N-A9KI-01A | 1 |
| TCGA-XF-A8HF-01A | 1 |
| TCGA-UY-A78L-01A | 2 |
| TCGA-FD-A5C0-01A | 1 |
| TCGA-UY-A9PE-01A | 2 |
| TCGA-K4-A6FZ-01A | 2 |
| TCGA-XF-A8HD-01A | 1 |
| TCGA-LC-A66R-01A | 1 |
| TCGA-GU-A762-01A | 1 |
| TCGA-XF-A9T5-01A | 1 |
| TCGA-4Z-AA7O-01A | 1 |
| TCGA-DK-A6B5-01A | 1 |
| TCGA-C4-A0F0-01A | 1 |
| TCGA-G2-A3IB-01A | 2 |
| TCGA-G2-AA3F-01A | 1 |
| TCGA-ZF-AA53-01A | 1 |
| TCGA-LT-A8JT-01A | 1 |
| TCGA-XF-A8HI-01A | 1 |
| TCGA-FJ-A3ZE-01A | 1 |
| TCGA-4Z-AA82-01A | 1 |
| TCGA-DK-AA6X-01A | 1 |
| TCGA-XF-A9SJ-01A | 1 |
| TCGA-CF-A7I0-01A | 1 |
| TCGA-GD-A3OQ-01A | 1 |
| TCGA-CU-A0YO-01A | 1 |
| TCGA-DK-AA71-01A | 1 |
| TCGA-CF-A9FL-01A | 1 |
| TCGA-ZF-AA5N-01A | 1 |
| TCGA-UY-A9PF-01A | 1 |
| TCGA-UY-A78P-01A | 2 |
| TCGA-CF-A1HR-01A | 1 |
| TCGA-DK-A3IN-01A | 1 |
| TCGA-XF-AAMG-01A | 1 |
| TCGA-GD-A76B-01A | 1 |
| TCGA-ZF-AA4U-01A | 1 |
| TCGA-ZF-AA4V-01A | 1 |
| TCGA-DK-AA6R-01A | 1 |
| TCGA-FT-A61P-01A | 1 |
| TCGA-2F-A9KQ-01A | 1 |
| TCGA-K4-A54R-01A | 1 |
| TCGA-BT-A20W-01A | 1 |
| TCGA-ZF-A9R3-01A | 1 |
| TCGA-BT-A42F-01A | 2 |
| TCGA-K4-A83P-01A | 1 |
| TCGA-4Z-AA80-01A | 1 |
| TCGA-XF-A9SX-01A | 1 |
| TCGA-KQ-A41N-01A | 1 |
| TCGA-FD-A3SM-01A | 2 |
| TCGA-DK-A3WX-01A | 1 |
| TCGA-DK-A3WW-01A | 1 |
| TCGA-XF-A9SK-01A | 1 |
| TCGA-FD-A3B7-01A | 2 |
| TCGA-CF-A3MG-01A | 1 |
| TCGA-CU-A0YR-01A | 2 |
| TCGA-UY-A78M-01A | 2 |
| TCGA-FD-A3SN-01A | 1 |
| TCGA-ZF-A9RN-01A | 1 |
| TCGA-4Z-AA81-01A | 1 |
| TCGA-E7-A8O8-01A | 2 |
| TCGA-GC-A3RB-01A | 1 |
| TCGA-BT-A20O-01A | 1 |
| TCGA-G2-A2ES-01A | 2 |
| TCGA-DK-AA6P-01A | 1 |
| TCGA-FD-A43P-01A | 1 |
| TCGA-E7-A678-01A | 1 |
| TCGA-2F-A9KT-01A | 2 |
| TCGA-CF-A9FF-01A | 1 |
| TCGA-UY-A9PA-01A | 1 |
| TCGA-ZF-AA58-01A | 2 |
| TCGA-2F-A9KW-01A | 2 |
| TCGA-4Z-AA87-01A | 1 |
| TCGA-GU-A42Q-01A | 2 |
| TCGA-HQ-A5ND-01A | 1 |
| TCGA-UY-A78K-01A | 1 |
| TCGA-UY-A9PH-01A | 1 |
| TCGA-BT-A3PH-01A | 2 |
| TCGA-ZF-AA4R-01A | 2 |
| TCGA-ZF-AA5P-01A | 1 |
| TCGA-DK-A3X2-01A | 1 |
| TCGA-YC-A8S6-01A | 1 |
| TCGA-FD-A3B5-01A | 1 |
| TCGA-GC-A3RC-01A | 2 |
| TCGA-BT-A0S7-01A | 1 |
| TCGA-GU-AATP-01A | 2 |
| TCGA-CF-A1HS-01A | 1 |
| TCGA-CF-A3MH-01A | 1 |
| TCGA-GC-A4ZW-01A | 1 |
| TCGA-ZF-A9RD-01A | 1 |
| TCGA-GU-A766-01A | 1 |
| TCGA-G2-A2EF-01A | 1 |
| TCGA-FD-A5BY-01A | 2 |
| TCGA-CF-A8HX-01A | 1 |
| TCGA-GV-A6ZA-01A | 1 |
| TCGA-FD-A3B4-01A | 1 |
| TCGA-4Z-AA7N-01A | 1 |
| TCGA-GC-A3RD-01A | 2 |
| TCGA-HQ-A2OE-01A | 1 |
| TCGA-FD-A5BZ-01A | 2 |
| TCGA-BT-A3PJ-01A | 2 |
| TCGA-DK-AA6L-01A | 2 |
| TCGA-CU-A72E-01A | 2 |
| TCGA-FD-A6TH-01A | 2 |
| TCGA-GD-A3OS-01A | 2 |
| TCGA-DK-A3IS-01A | 1 |
| TCGA-K4-A3WS-01A | 1 |
| TCGA-FD-A6TF-01A | 1 |
| TCGA-4Z-AA7R-01A | 1 |
| TCGA-DK-A6B1-01A | 1 |
| TCGA-E5-A4TZ-01A | 2 |
| TCGA-DK-A6AW-01A | 1 |
| TCGA-FD-A62S-01A | 2 |
| TCGA-DK-A3IU-01A | 1 |
| TCGA-ZF-AA4W-01A | 2 |
| TCGA-GC-A3YS-01A | 1 |
| TCGA-4Z-AA7Q-01A | 1 |
| TCGA-LT-A5Z6-01A | 2 |
| TCGA-GC-A3I6-01A | 1 |
| TCGA-E7-A5KF-01A | 1 |
| TCGA-DK-A3IM-01A | 2 |
| TCGA-FJ-A3Z9-01A | 2 |
| TCGA-CF-A47X-01A | 1 |
| TCGA-BT-A2LA-01A | 2 |
| TCGA-K4-A4AC-01A | 1 |
| TCGA-XF-AAMX-01A | 1 |
| TCGA-ZF-A9R5-01A | 1 |
| TCGA-XF-A8HC-01A | 1 |
| TCGA-ZF-A9RF-01A | 1 |
| TCGA-H4-A2HO-01A | 2 |
| TCGA-BT-A42E-01A | 1 |
| TCGA-FD-A5BU-01A | 1 |
| TCGA-XF-A9T6-01A | 1 |
| TCGA-ZF-AA4T-01A | 2 |
| TCGA-CF-A47W-01A | 1 |
| TCGA-K4-A5RJ-01A | 1 |
| TCGA-XF-A9SW-01A | 1 |
| TCGA-XF-AAMR-01A | 1 |
| TCGA-DK-AA6S-01A | 1 |
| TCGA-FD-A62P-01A | 2 |
| TCGA-FD-A6TC-01A | 2 |
| TCGA-XF-AAN1-01A | 1 |
| TCGA-GV-A3JV-01A | 1 |
| TCGA-FD-A43S-01A | 1 |
| TCGA-PQ-A6FN-01A | 2 |
| TCGA-K4-A5RI-01A | 1 |
| TCGA-ZF-A9R7-01A | 1 |
| TCGA-XF-A9SM-01A | 1 |
| TCGA-FD-A6TE-01A | 1 |
| TCGA-XF-AAME-01A | 1 |
| TCGA-BT-A42C-01A | 1 |
| TCGA-CF-A47Y-01A | 1 |
| TCGA-E7-A519-01A | 2 |
| TCGA-FD-A43Y-01A | 1 |
| TCGA-XF-A9SL-01A | 1 |
| TCGA-CF-A3MF-01A | 1 |
| TCGA-FD-A3B8-01A | 1 |
| TCGA-DK-A1A7-01A | 1 |
| TCGA-DK-A1A6-01A | 1 |
| TCGA-DK-A6B2-01A | 1 |
| TCGA-DK-AA74-01A | 1 |
| TCGA-ZF-AA4N-01A | 1 |
| TCGA-BT-A0YX-01A | 1 |
| TCGA-R3-A69X-01A | 1 |
| TCGA-XF-AAMW-01A | 1 |
| TCGA-C4-A0EZ-01A | 2 |
| TCGA-XF-A9T0-01A | 2 |
| TCGA-UY-A78N-01A | 1 |
| TCGA-E7-A7DU-01A | 1 |
| TCGA-DK-A3IT-01A | 1 |
| TCGA-MV-A51V-01A | 1 |
| TCGA-UY-A78O-01A | 2 |
| TCGA-FD-A6TK-01A | 1 |
| TCGA-4Z-AA83-01A | 1 |
| TCGA-BL-A0C8-01A | 1 |
| TCGA-DK-A2I6-01A | 1 |
| TCGA-XF-AAN4-01A | 1 |
| TCGA-CF-A27C-01A | 1 |
| TCGA-5N-A9KM-01A | 1 |
| TCGA-BT-A20U-01A | 2 |
| TCGA-YC-A9TC-01A | 1 |
| TCGA-E7-A6MD-01A | 1 |
| TCGA-K4-A5RH-01A | 1 |
| TCGA-XF-AAMH-01A | 2 |
| TCGA-DK-A1AB-01A | 1 |
| TCGA-GV-A3QI-01A | 1 |
| TCGA-FD-A43X-01A | 1 |
| TCGA-XF-A9SU-01A | 1 |
| TCGA-BT-A20J-01A | 1 |
| TCGA-E7-A4IJ-01A | 1 |
| TCGA-FD-A3B6-01A | 1 |
| TCGA-XF-A9SV-01A | 2 |
| TCGA-K4-AAQO-01A | 1 |
| TCGA-CU-A3YL-01A | 1 |
| TCGA-DK-A2I2-01A | 2 |
| TCGA-4Z-AA84-01A | 2 |
| TCGA-DK-A1AF-01A | 2 |
| TCGA-DK-AA6T-01A | 1 |
| TCGA-E5-A4U1-01A | 1 |
| TCGA-BT-A20Q-01A | 1 |
| TCGA-ZF-A9R4-01A | 1 |
| TCGA-KQ-A41Q-01A | 1 |
| TCGA-E7-A8O7-01A | 1 |
| TCGA-XF-AAN7-01A | 1 |
| TCGA-XF-A8HH-01A | 1 |
| TCGA-FD-A5BX-01A | 2 |
| TCGA-ZF-A9RE-01A | 1 |
| TCGA-FT-A3EE-01A | 1 |
| TCGA-XF-AAN8-01A | 1 |
| TCGA-XF-AAML-01A | 1 |
| TCGA-BT-A3PK-01A | 2 |
| TCGA-CF-A3MI-01A | 1 |
| TCGA-E7-A541-01A | 1 |
| TCGA-ZF-AA54-01A | 2 |
| TCGA-XF-AAMZ-01A | 1 |
| TCGA-CF-A8HY-01A | 1 |
| TCGA-DK-A6B6-01A | 1 |
| TCGA-DK-A6B0-01A | 1 |
| TCGA-E7-A97Q-01A | 1 |
| TCGA-K4-A3WV-01A | 1 |
| TCGA-E7-A85H-01A | 1 |
| TCGA-XF-A8HG-01A | 1 |
| TCGA-XF-A9T4-01A | 1 |
| TCGA-GD-A6C6-01A | 1 |
| TCGA-G2-A2EJ-01A | 2 |
| TCGA-UY-A9PD-01A | 2 |
| TCGA-E7-A677-01A | 1 |
| TCGA-FD-A3SQ-01A | 2 |
| TCGA-DK-AA77-01A | 1 |
| TCGA-K4-A6MB-01A | 2 |
| TCGA-ZF-AA51-01A | 2 |
| TCGA-E7-A7PW-01A | 1 |
| TCGA-KQ-A41P-01A | 2 |
| TCGA-G2-A3IE-01A | 1 |
| TCGA-E7-A5KE-01A | 1 |
| TCGA-FD-A6TG-01A | 1 |
| TCGA-2F-A9KO-01A | 1 |
| TCGA-CF-A47V-01A | 1 |
| TCGA-G2-AA3B-01A | 1 |
| TCGA-PQ-A6FI-01A | 1 |
| TCGA-C4-A0F7-01A | 1 |
| TCGA-FD-A5BS-01A | 1 |
| TCGA-ZF-A9RM-01A | 1 |
| TCGA-CU-A3KJ-01A | 2 |
| TCGA-XF-A9T8-01A | 1 |
| TCGA-FD-A5BR-01A | 1 |
| TCGA-4Z-AA7S-01A | 1 |
| TCGA-XF-A9SH-01A | 1 |
| TCGA-DK-A1AG-01A | 1 |
| TCGA-2F-A9KR-01A | 1 |
| TCGA-DK-AA6U-01A | 1 |
| TCGA-C4-A0F6-01A | 1 |
| TCGA-CU-A0YN-01A | 2 |
| TCGA-FJ-A871-01A | 1 |
| TCGA-G2-A2EC-01A | 1 |
| TCGA-FD-A5BV-01A | 1 |
| TCGA-E7-A7XN-01A | 1 |
| TCGA-GU-A764-01A | 1 |
| TCGA-FJ-A3Z7-01A | 1 |
| TCGA-XF-A9SZ-01A | 2 |
| TCGA-GU-AATO-01A | 1 |
| TCGA-ZF-A9RC-01A | 2 |
| TCGA-ZF-A9R0-01A | 2 |
| TCGA-BL-A13I-01A | 1 |
| TCGA-4Z-AA7W-01A | 1 |
| TCGA-HQ-A5NE-01A | 1 |
| TCGA-E7-A3Y1-01A | 1 |
| TCGA-XF-AAN5-01A | 1 |
| TCGA-G2-AA3D-01A | 1 |
| TCGA-GU-A42R-01A | 1 |
| TCGA-XF-A9SI-01A | 1 |
| TCGA-GV-A3JZ-01A | 1 |

**Supplementary Table 3** The proportion of immune cells in TMEscore-high and TMEscore-low groups.

|  | Proportion (%) | | P value |
| --- | --- | --- | --- |
|  | TMEscore-high | TMEscore-low |  |
| Dendritic cells activated | 7.48 | 2.28 | <0.001 |
| Monocytes | 3.34 | 1.63 | <0.001 |
| T cells follicular helper | 3.10 | 1.79 | 0.011 |
| T cells regulatory (Tregs) | 2.04 | 0.36 | <0.001 |
| Macrophages M2 | 11.72 | 16.16 | <0.001 |
| T cells CD4 memory resting | 11.00 | 13.17 | 0.043 |
| Macrophages M0 | 6.31 | 12.18 | <0.001 |
| Macrophages M1 | 1.15 | 6.47 | <0.001 |
| T cells CD8 | 6.16 | 5.68 | 0.840 |
| Mast cells resting | 3.96 | 3.18 | 0.076 |
| NK cells resting | 3.16 | 3.75 | 0.055 |
| B cells naive | 1.31 | 1.51 | 0.610 |
| Plasma cells | 0.68 | 0.25 | 0.260 |
| NK cells activated | 0.07 | 0.38 | 0.760 |
| B cells memory | 0.00 | 0.00 | <0.001 |
| T cells CD4 naive | 0.00 | 0.00 | <0.001 |
| T cells CD4 memory activated | 0.00 | 0.20 | <0.001 |
| T cells gamma delta | 0.00 | 0.00 | 0.057 |
| Dendritic cells resting | 0.00 | 0.00 | 0.024 |
| Mast cells activated | 0.00 | 0.00 | 0.890 |
| Eosinophils | 0.00 | 0.00 | <0.001 |
| Neutrophils | 0.00 | 0.00 | 0.048 |

**
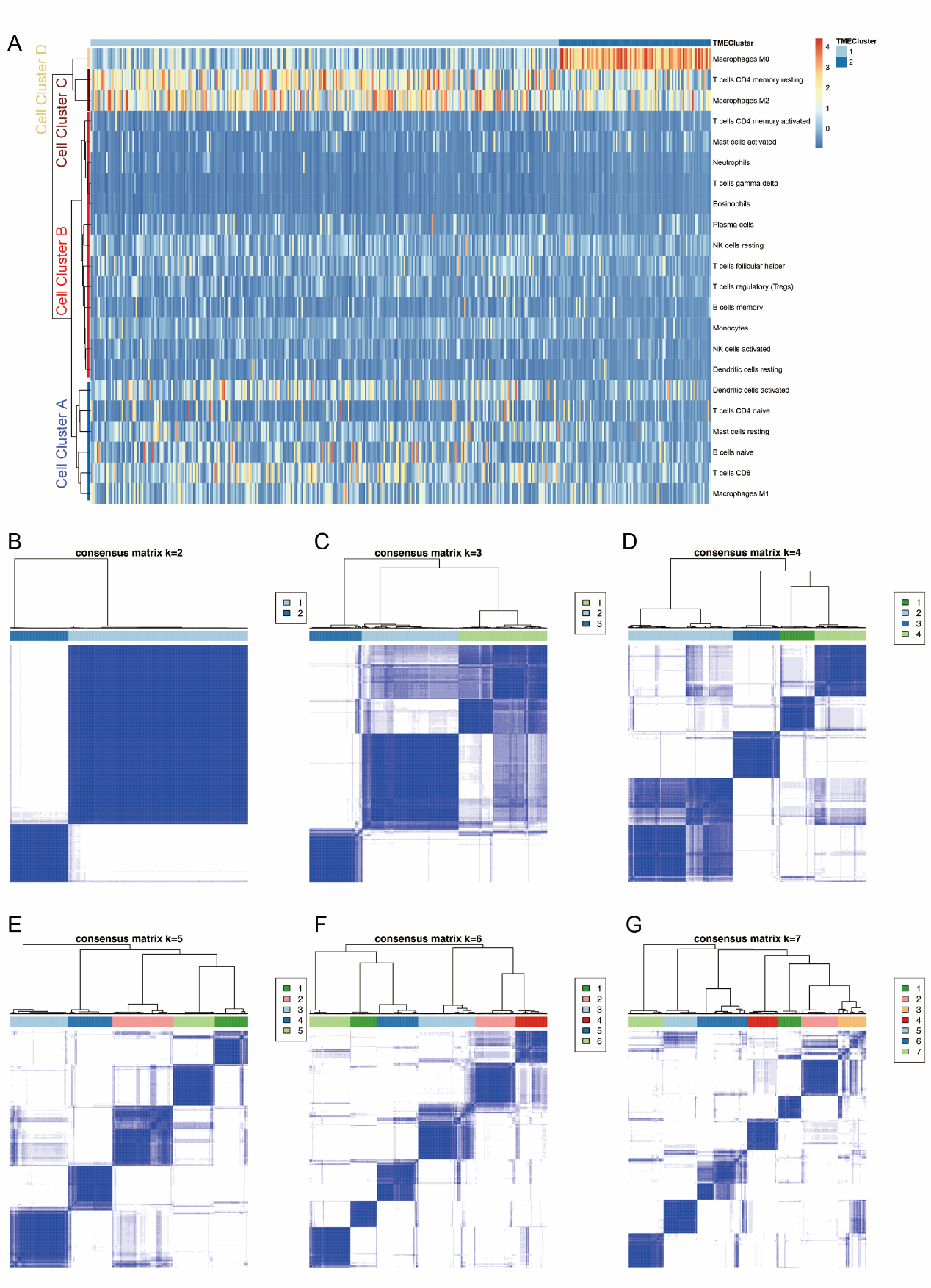

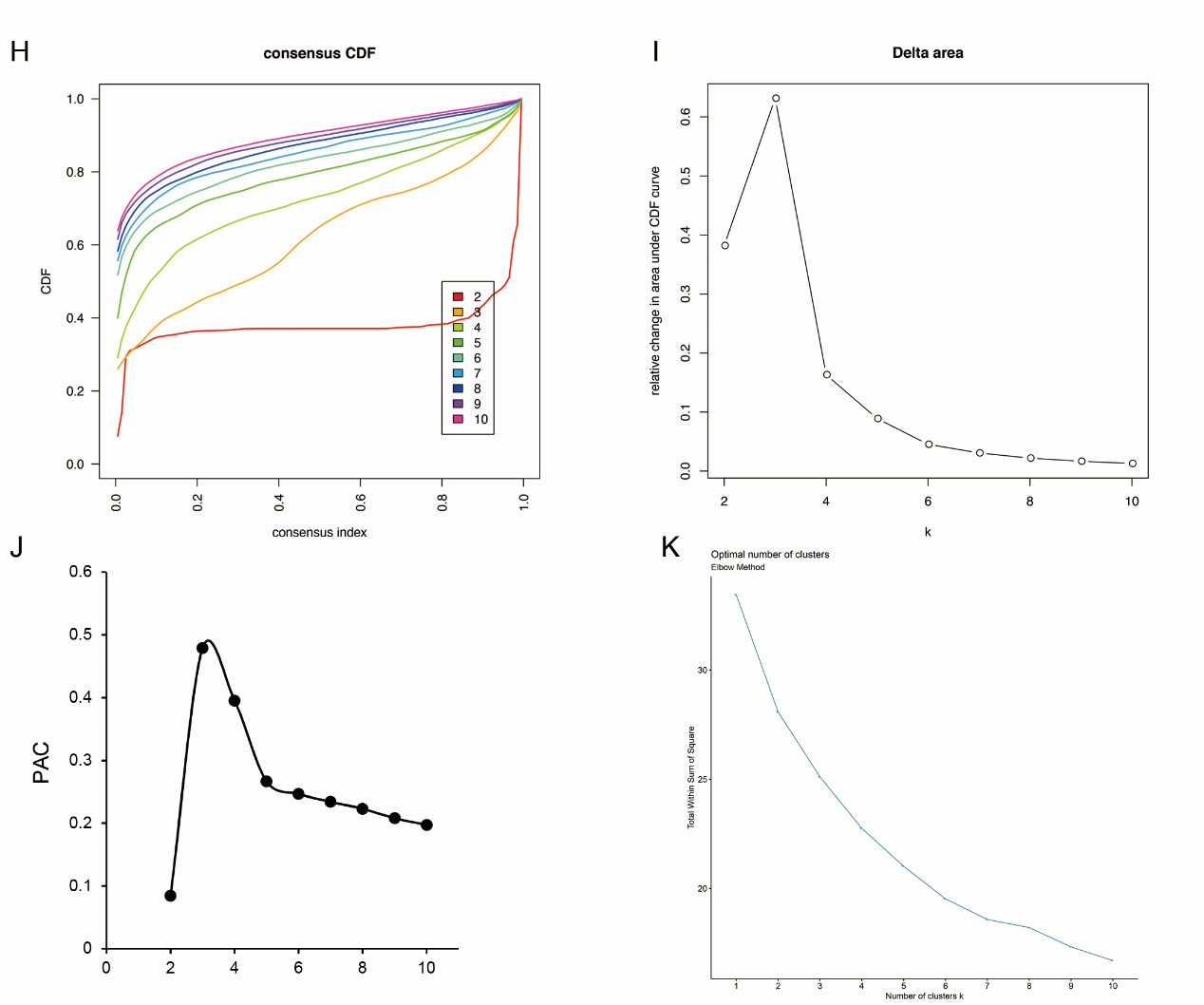

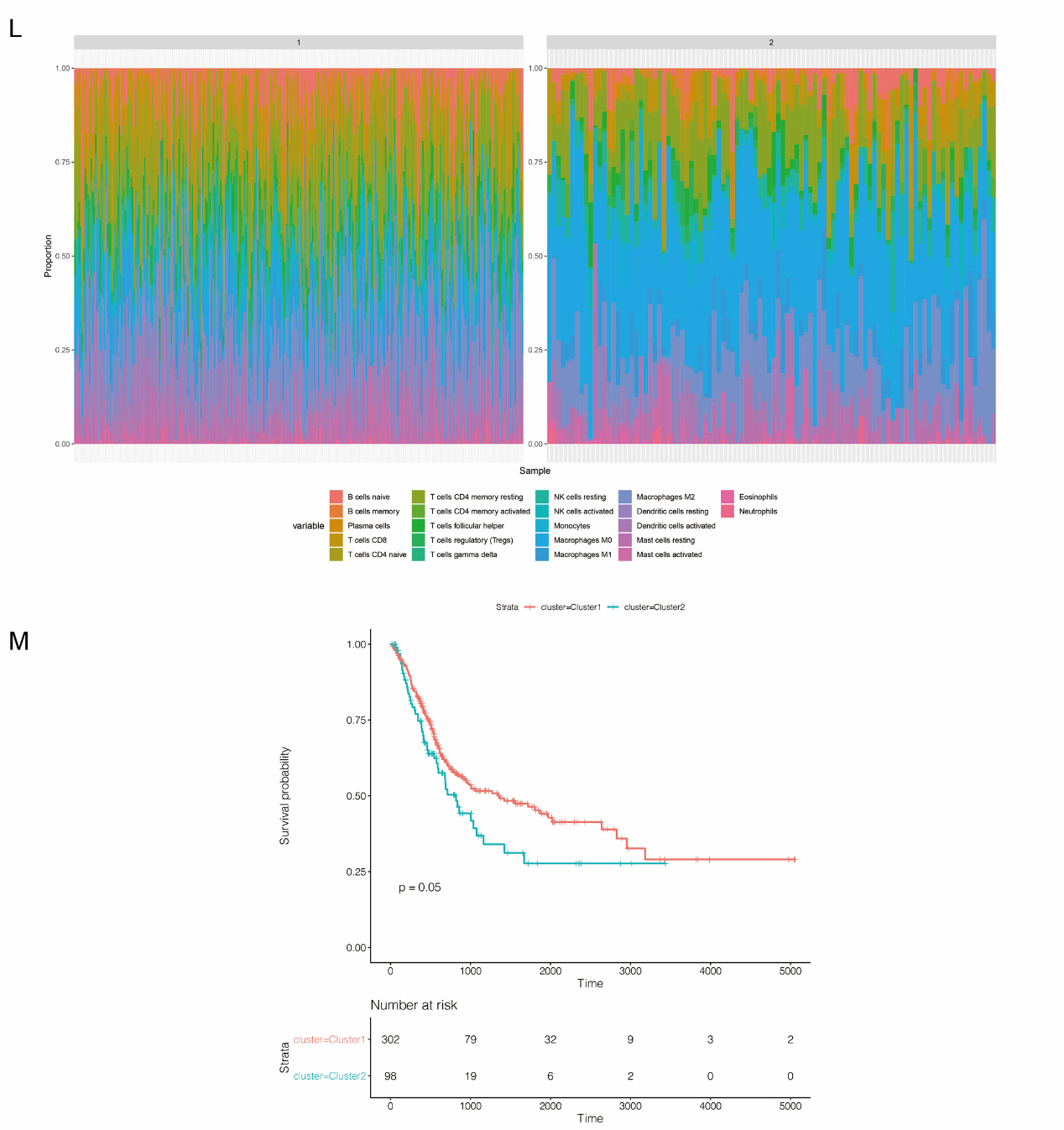
**

**Supplementary Figure 1.** The two TME clusters of BLCA based on the proportion of immune cells. (A) The hierarchical heatmap of different immune cells. (B)-(G) The heatmap of the consensus matrix. (H) The consensus CDF. (I) The delta area plot. (J) The PAC value. (K) The result of elbow method. (L) The proportions of 22 types of immune cells in two TME clusters. (M) The correlation of TME clusters with OS.


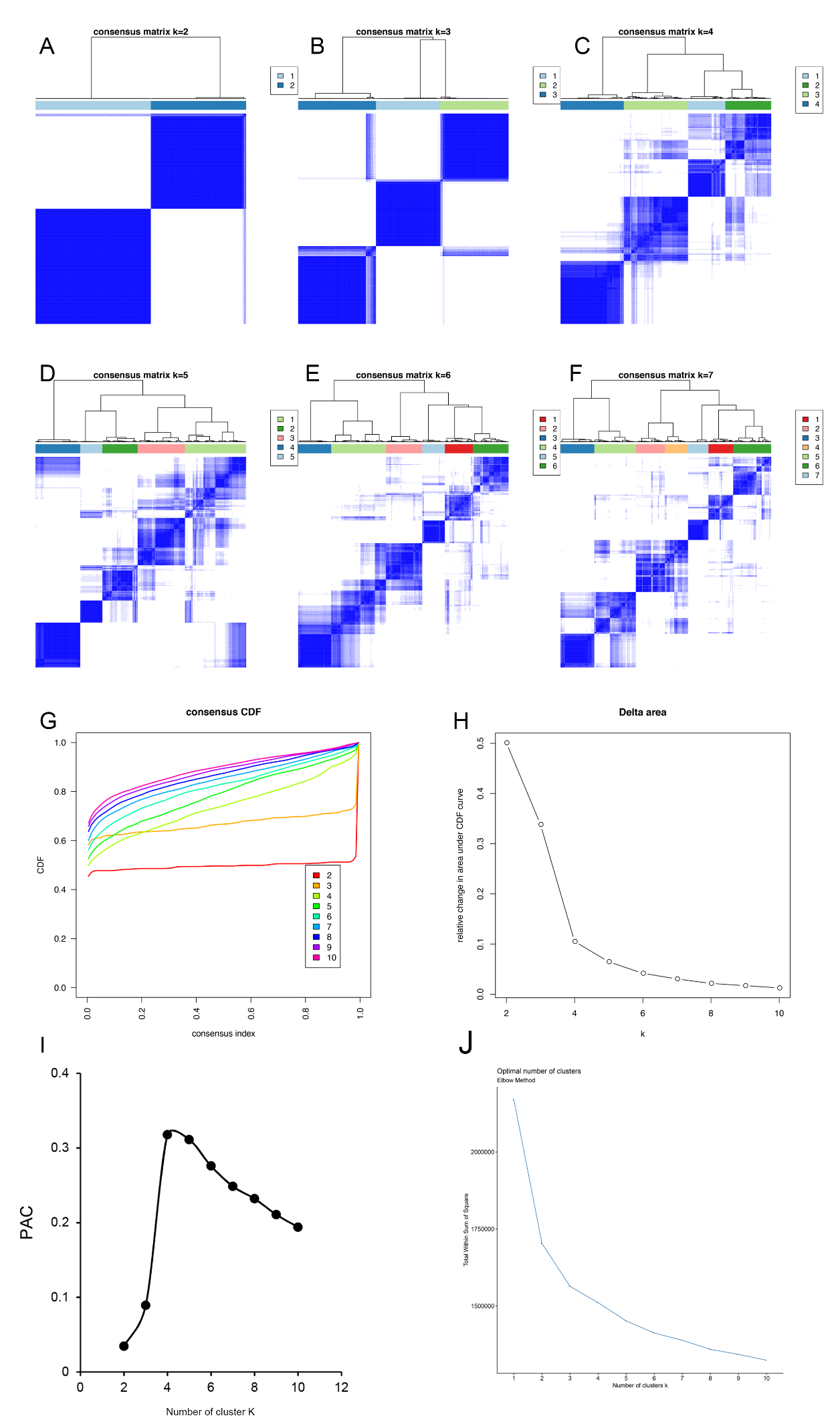


**Supplementary Figure 2.** The BLCA patients can be classified into three classes based on the DEGs between TMEclusters. (A)-(F) The heatmap of the consensus matrix. (G) The consensus CDF. (H) The delta area plot. (I) The PAC value. (J) The result of elbow method.


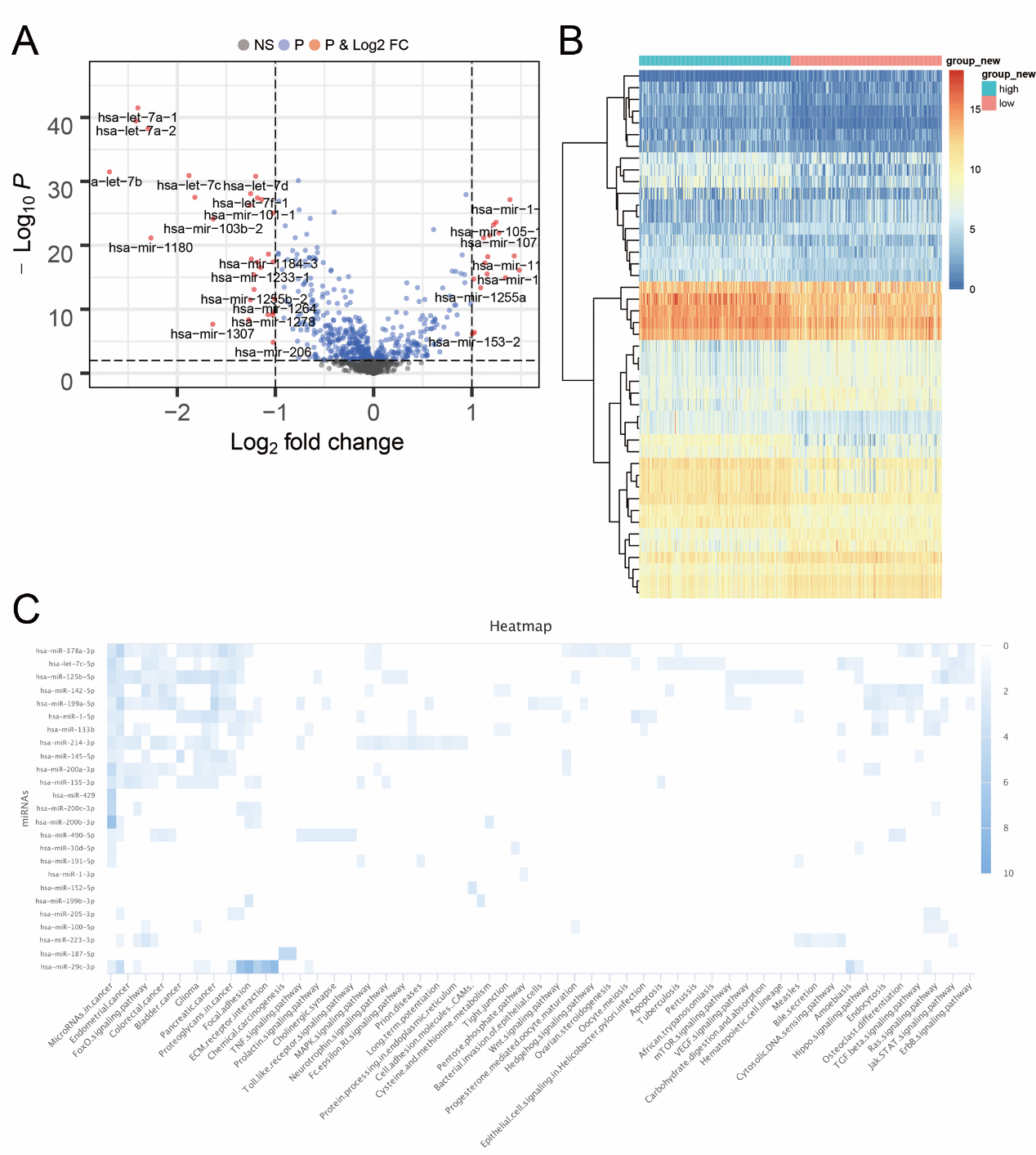


**Supplementary Figure 3.** Differentially expressed miRNAs between TMEscore-high and TMEscore-low subtypes. (A) The volcano plot. (B) The clustered heat map. (C) The enrichment analysis in KEGG.


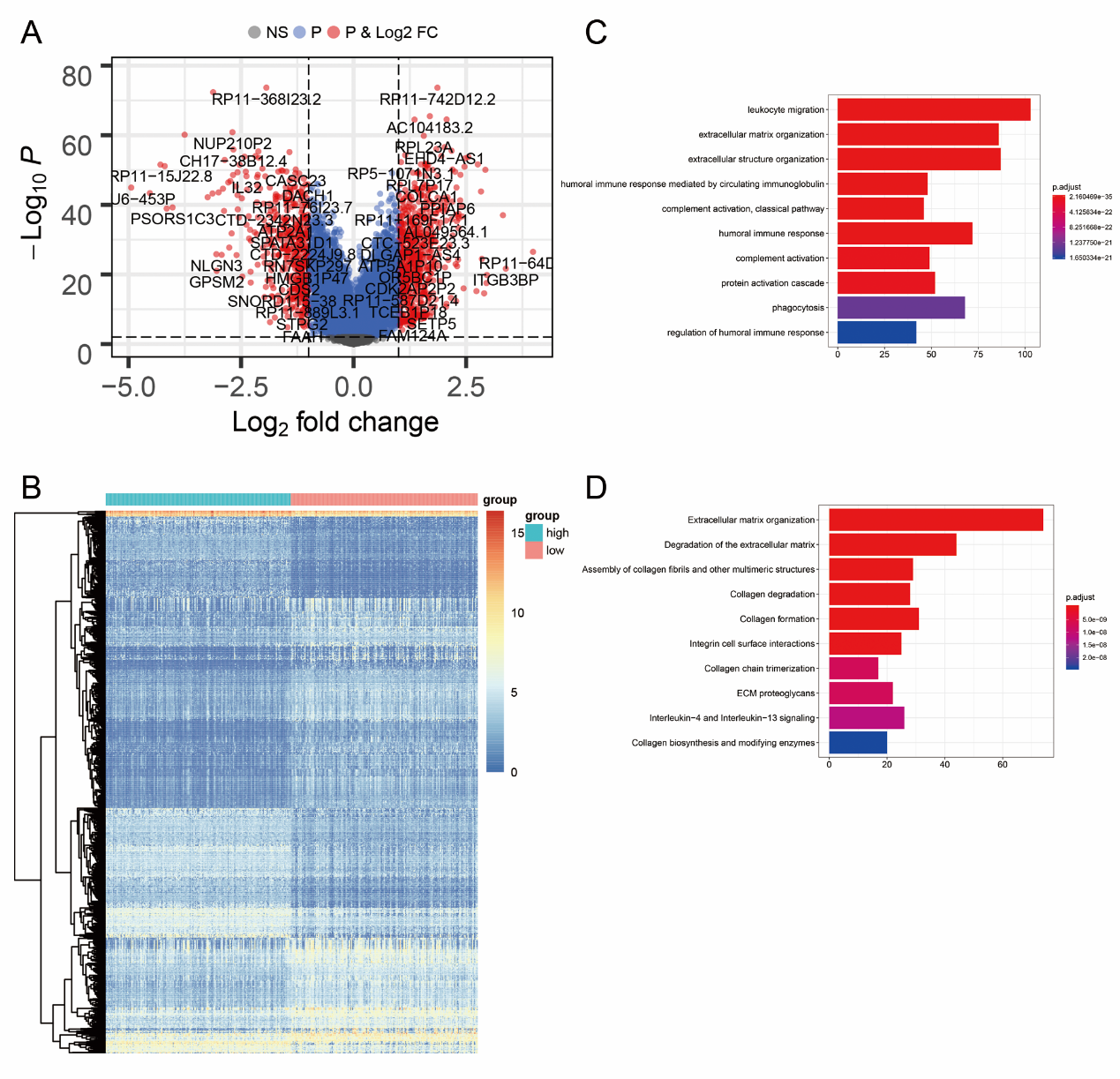
 **Supplementary Figure 4.** Differentially expressed mRNAs. (A) Volcano plot. (B) Clustered heat map. (C) GO enrichment analysis. (D) KEGG pathway analysis.

**
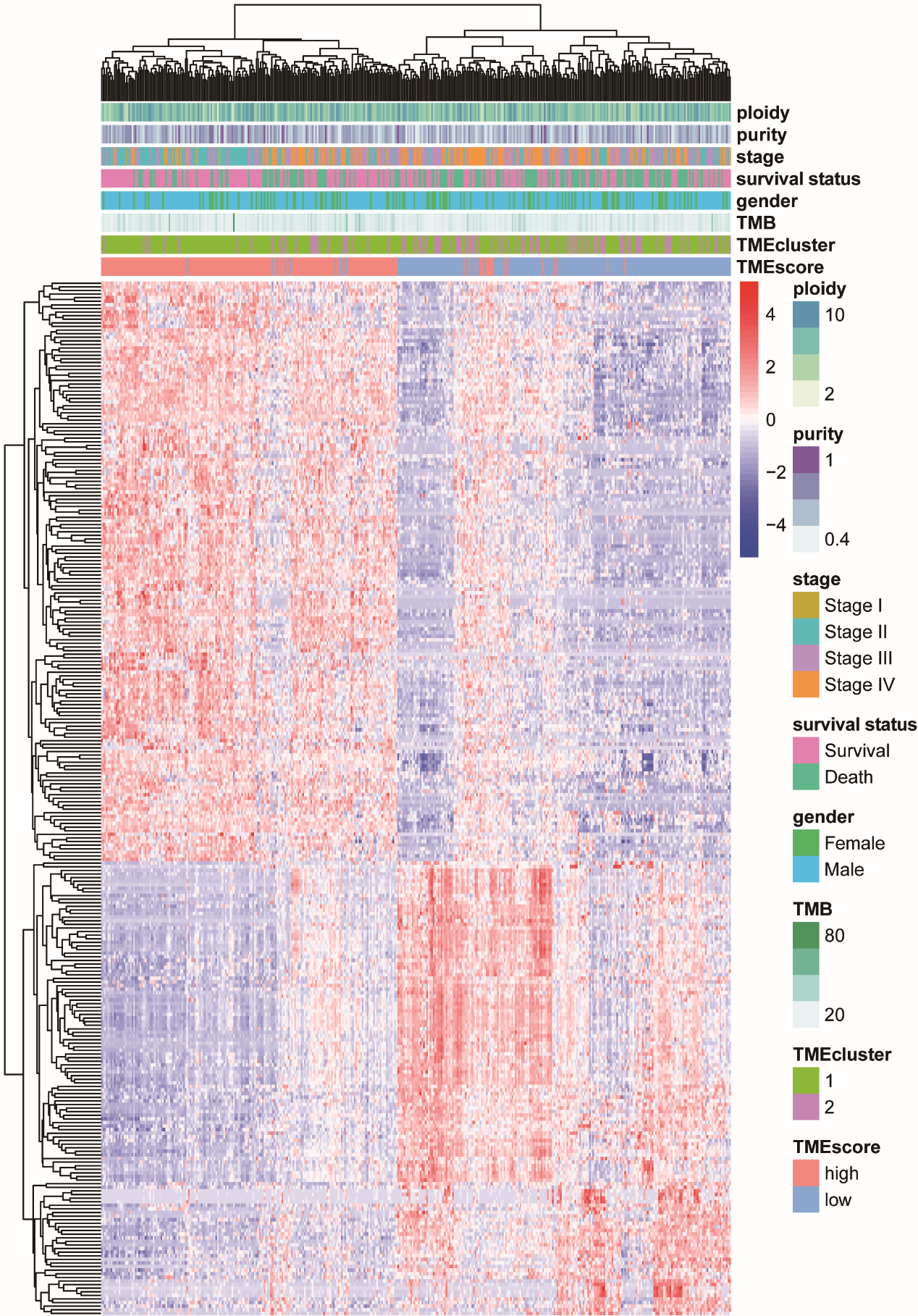
Supplementary Figure 5.** Unsupervised hierarchical clustering of 287 survival-related genes classified patients into TMEscore-high and TMEscore-low groups. Ploidy, purity, stage, survival status, gender, TMB and TMEcluster are shown as patient annotations.


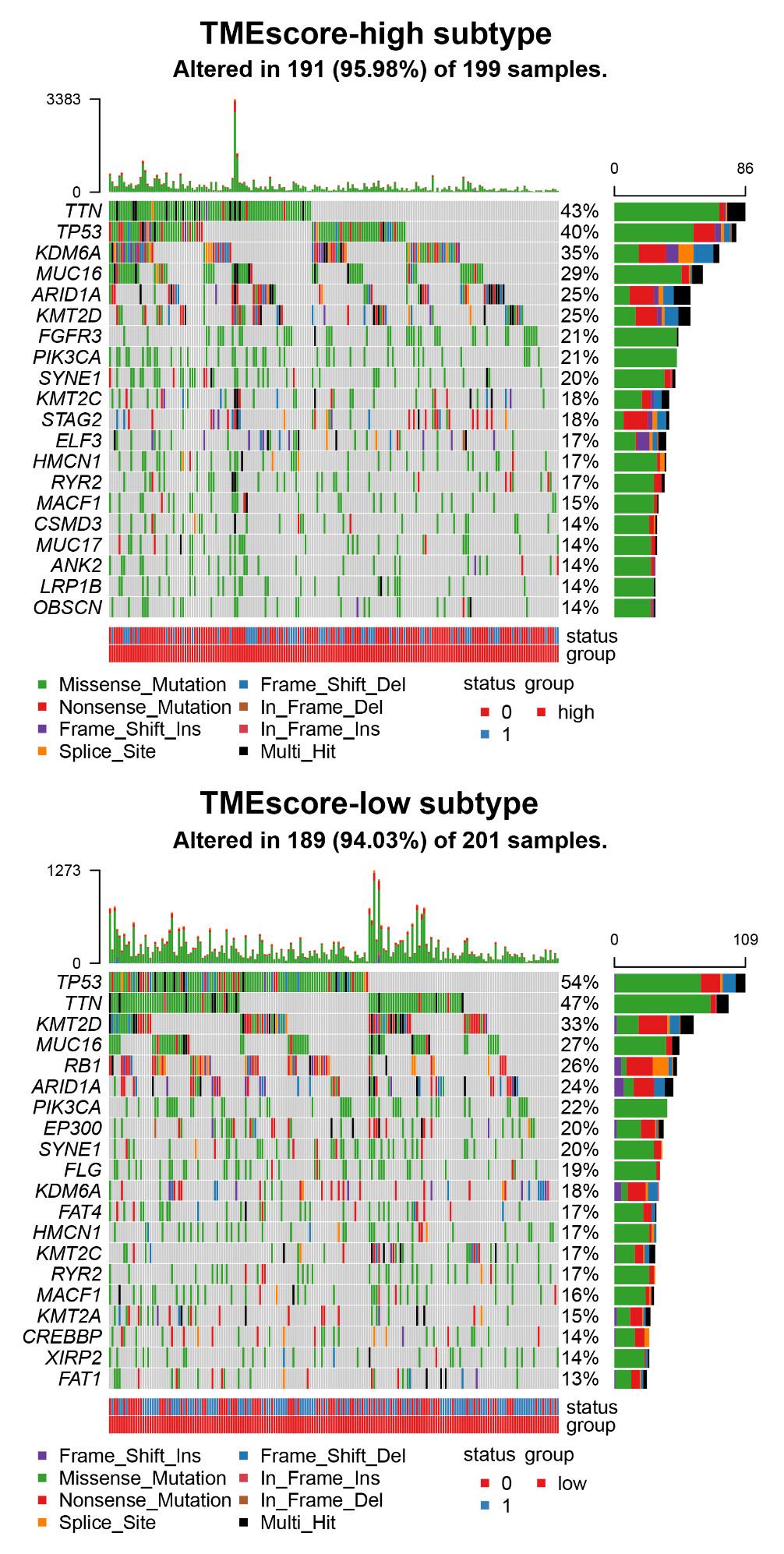


**Supplementary Figure 6.** The frequently mutated genes in TMEscore-high and TMEscore-low subtype.


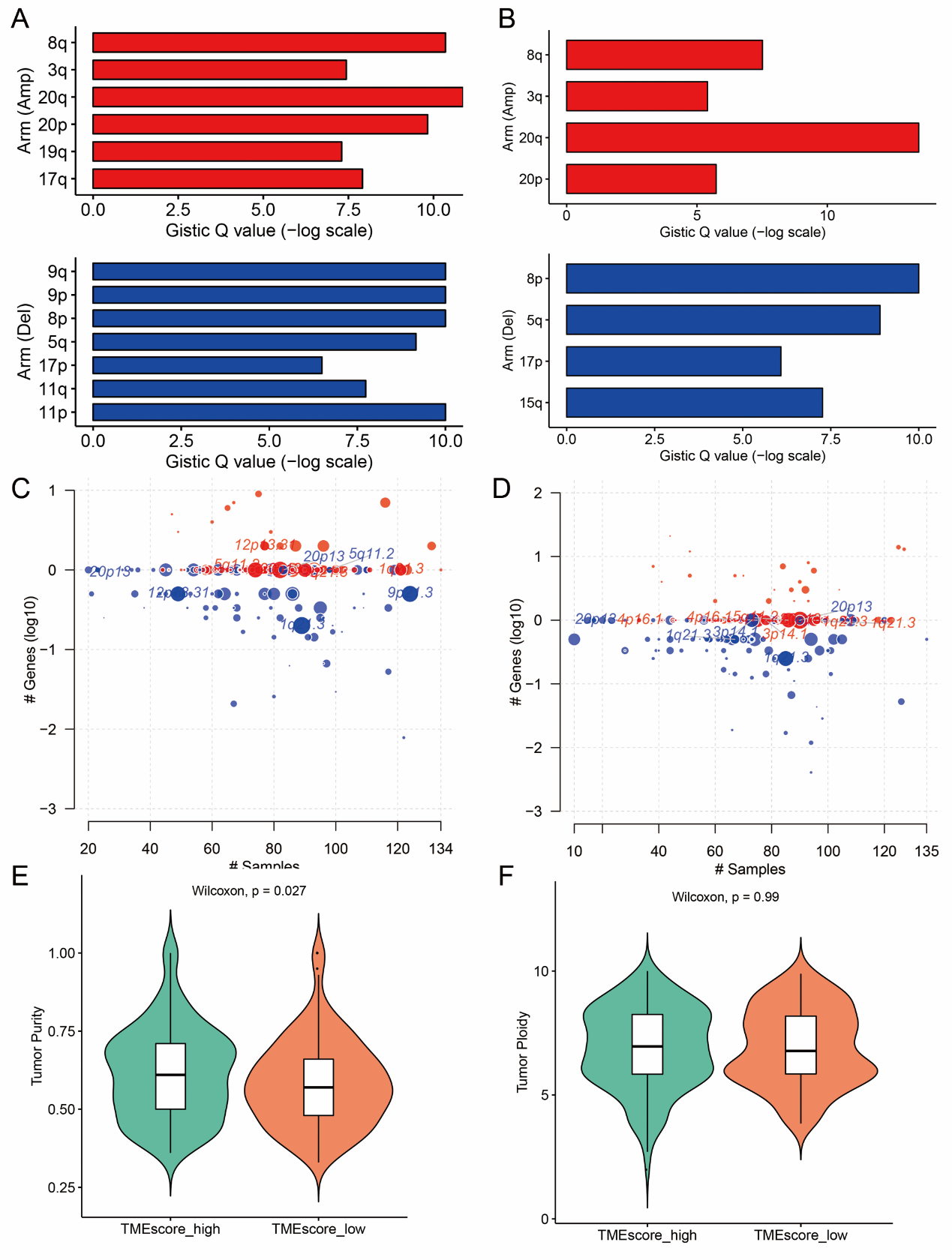
**Supplementary Figure 7.** The results of CNV analysis. The frequent amplifications (red bar) and deletions (blue bar) of chromosomal arms in TMEscore-high subtype (A) and in TMEscore-low subtype (B). The minimal common region (MCR) analysis in TMEscore-high subtype (C) and in TMEscore-low subtype (D). (E) The tumor purity in two TMEscore subtypes. (F) The tumor ploidy in two TMEscore subtypes.
